# Properties of beta oscillations in Dup15q syndrome

**DOI:** 10.1186/s11689-020-09326-1

**Published:** 2020-08-13

**Authors:** Vidya Saravanapandian, Joel Frohlich, Joerg F. Hipp, Carly Hyde, Aaron W. Scheffler, Peyman Golshani, Edwin H. Cook, Lawrence T. Reiter, Damla Senturk, Shafali S. Jeste

**Affiliations:** 1 Center for Autism Research and Treatment, Semel Institute for Neuroscience, University of California Los Angeles, Los Angeles, CA 90024 USA; 2 Roche Pharma Research and Early Development, Neuroscience, Ophthalmology and Rare Diseases, Roche Innovation Center Basel, Basel, Switzerland; 3 Department of Psychology, University of California Los Angeles, 3423 Franz Hall, Los Angeles, CA 90095 USA; 4 Department of Biostatistics, University of California Los Angeles School of Public Health, Room 21-254C CHS, Los Angeles, CA 90095 USA; 5 Department of Neurology and Semel Institute for Neuroscience, David Geffen School of Medicine, 710 Westwood Plaza, Los Angeles, CA 90095 USA; 6 West Los Angeles VA Medical Center, 11301 Wilshire Blvd, Los Angeles, CA 90073 USA; 7 Department of Psychiatry, University of Illinois at Chicago, 1747 W Roosevelt Road, Chicago, IL 60608 USA; 8 Department of Neurology, Pediatrics and Anatomy & Neurobiology, The University of Tennessee Health Science Center, 855 Monroe Ave., Link, Memphis, TN 415 USA

**Keywords:** Dup15q syndrome, Autism, Biomarkers, EEG, GABA, UBE3A, Neurodevelopmental disorders

## Abstract

**Background:**

Duplications of 15q11.2-q13.1 (Dup15q syndrome) are highly penetrant for autism, intellectual disability, hypotonia, and epilepsy. The 15q region harbors genes critical for brain development, particularly *UBE3A* and a cluster of gamma-aminobutyric acid type A receptor (GABA_A_R) genes. We recently described an electrophysiological biomarker of the syndrome, characterized by excessive beta oscillations (12–30 Hz), resembling electroencephalogram (EEG) changes induced by allosteric modulation of GABA_A_Rs. In this follow-up study, we tested a larger cohort of children with Dup15q syndrome to comprehensively examine properties of this EEG biomarker that would inform its use in future clinical trials, specifically, its (1) relation to basic clinical features, such as age, duplication type, and epilepsy; (2) relation to behavioral characteristics, such as cognition and adaptive function; (3) stability over time; and (4) reproducibility of the signal in clinical EEG recordings.

**Methods:**

We computed EEG power and beta peak frequency (BPF) in a cohort of children with Dup15q syndrome (*N* = 41, age range 9–189 months). To relate EEG parameters to clinical (study 1) and behavioral features (study 2), we examined age, duplication type, epilepsy, cognition, and daily living skills (DLS) as predictors of beta power and BPF. To evaluate stability over time (study 3), we derived the intraclass correlation coefficients (ICC) from beta power and BPF computed from children with multiple EEG recordings (*N* = 10, age range 18–161 months). To evaluate reproducibility in a clinical setting (study 4), we derived ICCs from beta power computed from children (*N* = 8, age range 19–96 months), who had undergone both research EEG and clinical EEG.

**Results:**

The most promising relationships between EEG and clinical traits were found using BPF. BPF was predicted both by epilepsy status (*R*^2^ = 0.11, *p* = 0.038) and the DLS component of the Vineland Adaptive Behavior Scale (*R*^2^ = 0.17, *p* = 0.01). Beta power and peak frequency showed high stability across repeated visits (beta power ICC = 0.93, BPF ICC = 0.92). A reproducibility analysis revealed that beta power estimates are comparable between research and clinical EEG (ICC = 0.94).

**Conclusions:**

In this era of precision health, with pharmacological and neuromodulatory therapies being developed and tested for specific genetic etiologies of neurodevelopmental disorders, quantification and examination of mechanistic biomarkers can greatly improve clinical trials. To this end, the robust beta oscillations evident in Dup15q syndrome are clinically reproducible and stable over time. With future preclinical and computational studies that will help disentangle the underlying mechanism, it is possible that this biomarker could serve as a robust measure of drug target engagement or a proximal outcome measure in future disease modifying intervention trials.

## Background

Genetic testing for neurodevelopmental disorders (NDDs) has become increasingly precise and clinically available. As a result, hundreds of causative genetic etiologies for NDDs have now been identified, from single gene mutations to copy number variants [1]. Under a conceptual framework of precision health for NDDs, identification of mechanistic biomarkers that reflect specific genetic disruptions can greatly improve clinical trials for these genetic syndromes by serving as measures of drug target engagement, stratification, or as outcome measures that precede more subtle, yet meaningful, behavioral responses to treatment [2, 3].

Recently, we quantified a robust electroencephalography (EEG) biomarker of the copy number variant syndrome caused by duplications of chromosome 15q11.2-q13.1 (Dup15q syndrome) [4–6]. Dup15q syndrome is highly penetrant for autism spectrum disorder (ASD), accounting for 1–3% of cases [7, 8]. Individuals with this syndrome also have comorbid global developmental delay, intellectual disability (ID), hypotonia, and a high rate of epilepsy [6, 9–11]. Based on allelic inheritance, the 15q region harbors several genes critical for brain development and synaptic function, particularly *UBE3A,* and a cluster of bi-allelically expressed gamma-aminobutyric acid type A receptor (GABA_A_R) genes, *GABRB3*, *GABRA5*, and *GABRG3,* which encode β3, α5, and γ3 subunits, respectively [10]. *UBE3A* encodes a ubiquitin protein ligase and is maternally expressed (i.e., paternally imprinted) in most neurons [12, 13] while playing an important role in regulating synaptic development and function [12, 14]. Functional loss of the *UBE3A* protein causes Angelman syndrome, another rare genetic NDD whose clinical features (e.g., ID and epilepsy) and etiology (e.g., *UBE3A* dysfunction) [15] partially overlap with Dup15q syndrome. However, the beta EEG phenotype found in cases of maternal Dup15q syndrome is also seen in paternal duplications, in which *UBE3A* is minimally impacted [5]. This suggests a crucial role for nonimprinted 15q genes, rather than *UBE3A*, in generating the EEG phenotype.

Different types of duplications result in Dup15q syndrome. Interstitial duplications generally result in one extra copy (i.e., partial trisomy) of the 15q region that remains on the same chromosome arm as the original copy. In some cases, interstitial triplications occur as two extra copies (i.e., partial tetrasomy) of the 15q region. Isodicentric duplications result in two extra maternal copies of the 15q region manifesting as a supernumerary chromosome [10]. Individuals with interstitial duplications tend to have a milder clinical phenotype and lower incidence of epilepsy compared to those with isodicentric duplications, implying a gene dosage effect [6, 7, 16] on clinical outcomes.

Spontaneous, high amplitude beta (12–30 Hz) oscillations represent an EEG biomarker of Dup15q syndrome [4–6]. This beta EEG phenotype was first noted in a comprehensive case series of children with interstitial duplications, based on clinical EEGs obtained for epilepsy monitoring [6, 17]. Our group then quantified this EEG phenotype in high-density research EEG recordings and found that beta oscillations significantly distinguish children with Dup15q syndrome from age-matched typically developing children and age- and cognitively matched children with nonsyndromic ASD and ID [4]. Evidence from clinical and preclinical studies demonstrates a crucial role for GABAergic neurotransmission in the generation of beta oscillations, thus implicating the GABAergic system in the Dup15q syndrome beta EEG phenotype. Positive allosteric modulators of GABA_A_Rs (e.g., benzodiazepines) induce beta oscillations in human scalp recordings and intracranial recordings from rodents [18–21]. These pharmacological agents enhance the inhibitory chloride current through the GABA_A_R when bound in the presence of GABA [22]. Conversely, blockade of GABA_A_Rs results in desynchronization and diminished oscillatory power at high frequencies in the beta/gamma (12–80 Hz) range [23]. Furthermore, cases of Angelman syndrome caused by deletions of 15q11-q13 (the genetic converse of Dup15q syndrome) demonstrate lower EEG beta power as compared with etiologies not involving the GABA_A_ β3/α5/γ3 gene cluster [24], also suggesting that beta power may serve as a biomarker of altered GABA neurotransmission.

Although an exact mechanism of beta oscillations has not been elucidated in Dup15q syndrome, increased gene dosage of *GABRB3*, *GABRA5*, and *GABRG3* [16, 25–27] suggests dysfunctional GABAergic neurotransmission. While the foregoing genes are likely crucial to the presence of the beta EEG phenotype [5], *UBE3A* plays a critical role in the development and function of GABAergic circuits, including neurons that co-release GABA in rodents [28, 29], suggesting that it also affects beta oscillations. As further evidence of the intimate connection between *UBE3A* and GABA, the GABA_A_ enhancers gaboxadol and ganaxolone have been shown to restore behavioral phenotypes in Ube3a knockout mice [30, 31]. Thus, *UBE3A* overexpression likely affects GABAergic transmission in this syndrome. Future studies focusing on quantifying beta oscillations in *UBE3A* overexpressed mouse models will help elucidate the exact relationship between *UBE3A* and beta oscillations in Dup15q syndrome.

As disease modifying therapies, particularly those that modulate altered GABA signaling, are developed and tested in Dup15q syndrome, the EEG biomarker in this syndrome has the potential to serve as a measure of drug target engagement, stratification, or as a proximal outcome measure. In order to facilitate and inform the use of this biomarker in clinical trials, we examined the following properties in a large cohort of children with this syndrome: (1) relation to clinical features, including age, duplication type, and epilepsy; (2) relation to behavior, namely those features that contribute most to the clinical heterogeneity of the syndrome (cognition and adaptive skills); (3) stability over time; and (4) reproducibility of the signal in clinical EEG. Given that this work spanned several years and projects, we also had the opportunity to compare results generated from different EEG data pre-processing pipelines, thus indirectly testing the reproducibility of the biomarker across analytic pipelines. To accomplish these goals and to adequately enhance our clinically representative sample size in this rare disorder, we partnered with a patient advocacy group, the Dup15q Alliance, and we collected EEG data at two consecutive national family meetings, as well as at our own institution. This study reflects an effort to improve clinical trial readiness in this genetic syndrome by comprehensively characterizing the aspects of this biomarker that would then guide its future use in treatment studies.

## Methods

### Sites for data collection

Data were collected at the University of California, Los Angeles (UCLA) and at two national Dup15q syndrome family conferences. In order to ensure that there were no site differences in EEG outcome, a one-way analysis of variance (ANOVA) with post-hoc tests was performed. There was no evidence of an effect of site on beta power (*F*(2, 38) = 0.28; *p* = 0.75) or beta peak frequencies (*F*(2, 38) = 1.10; *p* = 0.34).

### Participants

**Fig. 1:**
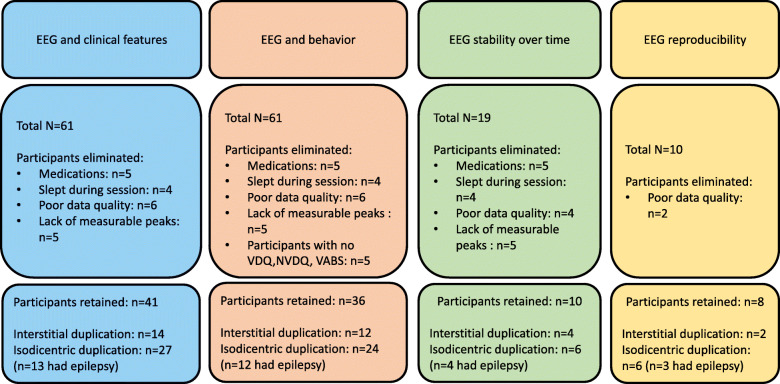
Flowchart showing participant distribution for each study

Children (age < 18 years) were clinically referred through the Dup15q clinic at UCLA and the Dup15q Alliance. Combining data collected from the three sites, EEG recordings were analyzed from a total of *n* = 61 participants. The flowchart in Fig. 1 shows the participant distribution for each parallel study described in this paper and reasons for exclusion from analysis.

### Behavioral assessments

Participants were administered the following measures: (1) The Mullen Scales of Early Learning (MSEL), which assesses general cognition and development [32]. The MSEL yields standard as well as age-equivalent scores that measure receptive and expressive language, visual reception, and gross and fine motor skills. Verbal and non-verbal cognition scores were then calculated. Given that most of our children with Dup15q syndrome had significant delays in overall development, age-equivalent ratio scores were used instead of standardized development quotient scores. (2) Vineland Adaptive Behavior Scale (VABS), a parent reported measure of adaptive behavior, which yields standard and age-equivalent scores for communication, daily living skills (DLS), and socialization and motor skills [33].

### EEG data acquisition

All data were collected under protocols approved by the Institutional Review Board (IRB) (# 15-001565). High-density research EEG data were acquired at a sampling rate of 500 Hz using 129 channel vertex-referenced Philips Neuro (Eugene, OR, USA) nets with Ag/AgCl electrodes. Full details of the research EEG acquisition can be found in a previous publication [4]. For the reproducibility analysis, we accessed overnight clinical EEG recordings that were collected at UCLA as part of routine epilepsy monitoring. These EEGs were collected at a sampling rate of 200 Hz using a 21-channel 10–20 montage data acquisition set up.

### EEG data elimination

We eliminated data from participants with (1) medications that are known to pharmacologically induce beta oscillations (benzodiazepines or barbiturates), (2) poor data quality due to artifacts from non-neural sources, or (3) lack of measurable peak (i.e., local maximum) in the beta band of the EEG power spectrum. Our final cohort of children with Dup15q syndrome yielded *n* = 41 participants. Details of age, sex, duplication type, epilepsy status, medications, and IQ can be found in Table 1.

**Table 1:** Dup15q syndrome participant characteristics (cross-sectional study)

Age (months)	Gender	Medications (generic)	Duplication Type	Epilepsy (active)	Verbal DQ	Non-verbal DQ	VABS_DLS
9	M	None	Interstitial	No	66.67	61.11	106.00
18	M	None	Isodicentric	No	50.00	52.78	77.00
29	M	Oxcarbazepine	Isodicentric	Yes	15.52	20.69	51.00
38	M	Levetiracetam	Isodicentric	Yes	13.16	25.00	38.00
39	M	None	Isodicentric	No	30.77	32.05	60.00
42	M	None	Isodicentric	No	N/A	N/A	57.00
44	M	None	Interstitial	No	91.00	68.00	83.00
46	F	None	Isodicentric	No	16.30	2.17	53.00
47	M	None	Isodicentric	No	73.40	69.15	83.00
48	M	None	Interstitial	No	N/A	N/A	66.00
50	F	None	Isodicentric	No	63.00	46.00	73.00
51	M	None	Isodicentric	No	32.35	38.24	54.00
53	M	Lamotrigine	Isodicentric	Yes	9.43	19.81	57.00
54	F	None	Interstitial	No	85.19	97.22	81.00
54	M	None	Interstitial	No	8.33	24.07	48.00
57	M	None	Isodicentric	No	47.37	44.74	N/A
61	F	Valproic acid, clobazam, lacosamide, perampanel	Isodicentric	Yes	9.02	13.93	50.00
62	F	Rufinamide, valproic acid	Isodicentric	No	50.00	30.65	57.00
64	M	Lamotrigine, rufinamide	Isodicentric	Yes	10.16	15.63	45.00
65	M	None	Isodicentric	Yes	9.23	13.08	38.00
67	F	None	Interstitial	No	37.31	37.31	57.00
68	F	None	Interstitial	No	14.71	21.32	N/A
69	M	Valproic acid, topiramate	Isodicentric	Yes	12.32	13.04	45.00
78	M	None	Interstitial	No	19.87	21.15	50.00
82	M	Valproic acid, oxcarbazepine	Isodicentric	Yes	N/A	N/A	36.00
94	M	None	Isodicentric	No	54.00	39.00	N/A
96	F	None	Isodicentric	No	N/A	N/A	61.00
100	M	Carbamazepine, levetiracetam	Isodicentric	Yes	54.00	64.00	66.00
102	F	None	Isodicentric	No	34.31	22.55	58.00
106	M	None	Interstitial	No	N/A	N/A	71.00
108	M	None	Interstitial	No	64.35	60.65	73.00
111	F	None	Interstitial	No	26.58	23.87	55.00
118	F	None	Interstitial	No	36.86	44.07	N/A
127	F	Topiramate	Isodicentric	Yes	22.83	20.47	47.00
134	M	None	Isodicentric	No	9.70	18.66	38.00
153	M	Rufinamide, levetiracetam, lacosamide, epidolex	Isodicentric	Yes	24.18	22.22	N/A
156	M	None	Isodicentric	No	37.18	24.20	40.00
161	M	None	Interstitial	No	73.00	66.00	73.00
169	M	Rufinamide, valproic acid	Isodicentric	Yes	16.57	13.02	20.00
175	F	None	Interstitial	No	31.00	41.00	86.00
189	M	None	Isodicentric	Yes	40.00	36.00	82.00

Cognitive tests were not available for all the participants. *N/A* not available. Dosages were not available for all the medications listed, hence not included in the table

Of the participants that had at least two research EEG recordings from multiple visits, 4 had recordings from three visits. We selected recordings that were at least 10 months apart to enforce this duration of time as a buffer between repeated observations. A total of 10 participants were included in the longitudinal stability analysis. See Table 2 for details of age, epilepsy status (at the time of visit), and duplication type. Dosages of medications were not confirmed with each participant’s physician and, therefore, were not included in the table. A subset of our sample (*n* = 8) had additional EEG recorded in a clinical setting for epilepsy monitoring.

**Table 2:** Dup15q syndrome participant characteristics (longitudinal study)

Participant	Duplication type	Visit 1 Age (months)	Visit 2 Age (months)	Epilepsy (active)	Medications
P1	Isodicentric	57	72	No	N/A
P2	Isodicentric	18	28	Yes (at second visit)	Vigabatrin (at second visit)
P3	Interstitial	51	78	No	N/A
P4	Isodicentric	110	134	No	N/A
P5	Isodicentric	84	100	Yes (at both visits)	Carbamazepine; levetiracetam (unchanged at both visits)
P6	Interstitial	48	72	No	N/A
P7	Isodicentric	29	53	Yes (at second visit)	Lamotrigine (at second visit)
P8	Isodicentric	38	61	Yes (at both visits)	Valproate, clobazam, lacosamide, perampanel (unchanged at both visits)
P9	Interstitial	44	68	No	N/A
P10	Interstitial	161	185	No	N/A

*N/A* not available. Dosages were not available for all the medications listed, hence not included in the table

### EEG Data processing

Data collection for the described studies spanned several years, and data were processed at two different sites (site 1, Hoffmann-La Roche, Ltd., Basel, Switzerland; site 2, UCLA, Los Angeles, USA) with two different data processing pipelines. To confirm the consistency of the quantified biomarker between different analytic approaches, we compared beta power and peak frequency estimates from data that were processed through both pipelines. Results showed strong reproducibility between analytic pipelines and encouraged us to use the same descriptions of EEG variables (“beta power” and “beta peak frequency”) for output from both pipelines despite differences in processing (see Results section for details). Of note, processing methods remained consistent within each study aim (i.e., data from different pipelines were not mixed within one analysis).

For studies 1 and 2 (relation to clinical and behavioral features), EEG data were analyzed at site 1 as an extension of recent work elucidating the mechanism of the EEG biomarker [5] using a combination of in-house tools and the MATLAB software toolbox Fieldtrip [34]. EEG signals were bandpass filtered 1–45 Hz using a finite impulse response filter (FIR). Sections of data containing gross artifacts and noisy channels were identified by visual inspection and excluded from analysis. Next, noisy channels were marked bad and excluded from subsequent independent component analysis (ICA); a statistical blind source separation technique was implemented to remove physiological artifacts including eye blinks, saccades, ballistocardiogram, and muscle activity, using the Fast ICA algorithm [35, 36]. Finally, rejected channels were spatially interpolated and data were re-referenced to average (in all studies, datasets were discarded when the number of bad channels exceeded the square root of the total number of channels). To derive spectral power estimates, logarithmically scaled frequencies with a spectral smoothing using Morlet wavelets were employed (2 to 45 Hz, 12 wavelets per octave) [37]. Power was then averaged across successive 3/4-overlapping temporal windows of continuous clean data after discarding time points corresponding to artifacts. Next, power was normalized with respect to the log_2_(frequency), resulting in a power spectral density (PSD) with units of μV2/log2(Hz) (i.e., power per octave) rather than μV2/Hz, thus accounting for the logarithmic nature of electrophysiological signals [38]. We reported beta power using trapezoidal integration in the 12–30 Hz band (MATLAB: trapz, absolute power integrated with respect to log_2_(frequency)). For further details of data processing see Frohlich et al. [5]

For studies 3 and 4, a separate data processing pipeline was applied using the EEGLAB software toolbox [39] for MATLAB. In this pipeline, data were FIR filtered 1–45 Hz. Sections of data containing gross artifacts and noisy channels were identified by visual inspection and excluded from analysis. Data were interpolated to a 25-channel montage before using ICA (infomax algorithm; EEGLAB: “runica”) to remove physiological artifacts. Data were then re-referenced to an average of all channels. For each electrode, PSDs were computed according to Welch’s method [4, 40], with power normalized per Hz (yielding μV2/Hz). Beta power was reported as the sum of the absolute power in the 12–30 Hz band.

In order to extract peak frequencies within the beta band, power spectra computed using aforementioned methods were averaged across electrodes. Peak labeling was performed automatically using the local maximum in the beta band, as well as manually using visual inspection. Manual labeling was performed by two trained raters. Raters were blinded to epilepsy status. In instances of manual labeling, an average of the peak labeling values obtained from the two raters was used. For each participant, automatically and manually labeled peak frequencies were compared. When the automated peak labeling fell within 5% of the value of the manual peak labeling, values from automated labeling were used. Otherwise, values from manual labeling were used.

### Data analysis

In order to ensure that there were no differences in the dependent variables between the two data processing methods, we compared recordings from participants (*n* = 8) that were processed using both pipelines, and intraclass correlation coefficients (ICC) were derived.

#### Studies 1 and 2: Relation to clinical and behavior features

To determine the relation between EEG and clinical features, simple linear regression models were performed using beta power and BPF as outcome measures, and duplication type, epilepsy status, and age as separate predictors of beta power and BPF. Age was treated as a continuous variable, while duplication type and epilepsy were treated as binary variables. Next, to determine the relationship between EEG and behavior, beta power and peak frequency were regressed on quantitative measures of cognition, as well as parent reported measures of social skills.

#### Studies 3 and 4: EEG stability and reproducibility

To evaluate stability of spectral power and peak frequency in the beta band across time points, ICCs were derived from EEG recordings of all participants with more than one research visit. To evaluate reproducibility of the EEG signature in Dup15q syndrome, beta power was compared between (a) high-density research EEG recorded from participants while they were awake and resting, and (b) low-density clinical EEG collected for epilepsy monitoring, with data extracted from segments in which participants were awake and resting prior to entering sleep. Data from low-density clinical EEG and high-density research EEG was computed and compared through derived ICCs.

Because our studies were performed in parallel, with no primary analysis selected between the four, we did not correct for multiple comparisons within or across the studies. Ninety-five percent confidence intervals (CIs) and effect sizes (where applicable) are reported to allow the reader to better interpret the meaningfulness of each finding.

## Results

The comparison of the two different pre-processing pipelines (site 1 and site 2) yielded an ICC of 0.93 (95% confidence interval 0.67–0.99) for beta power and an ICC of 0.92 (95% confidence interval 0.64–0.98) for BPF, indicating moderate to excellent correlation between the two data processing methods.

### Study 1: Relation to clinical features (age, duplication type, and epilepsy)

**Fig. 2:**
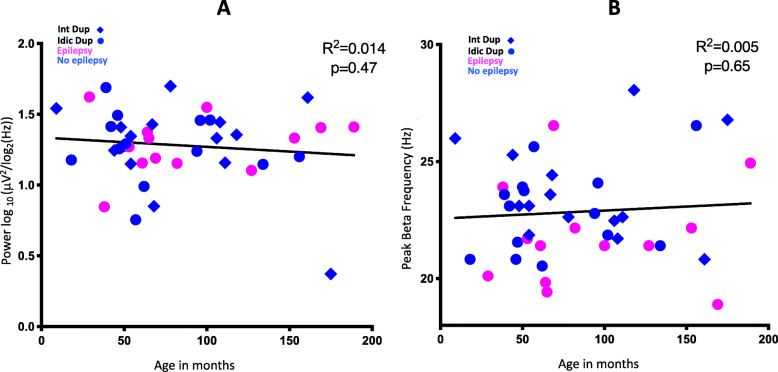
Age and beta power/beta peak frequency. **a** Age vs. beta power. **b** Age vs. beta peak frequency. Participants with epilepsy are shown in pink and those without epilepsy in blue

**Fig. 3:**
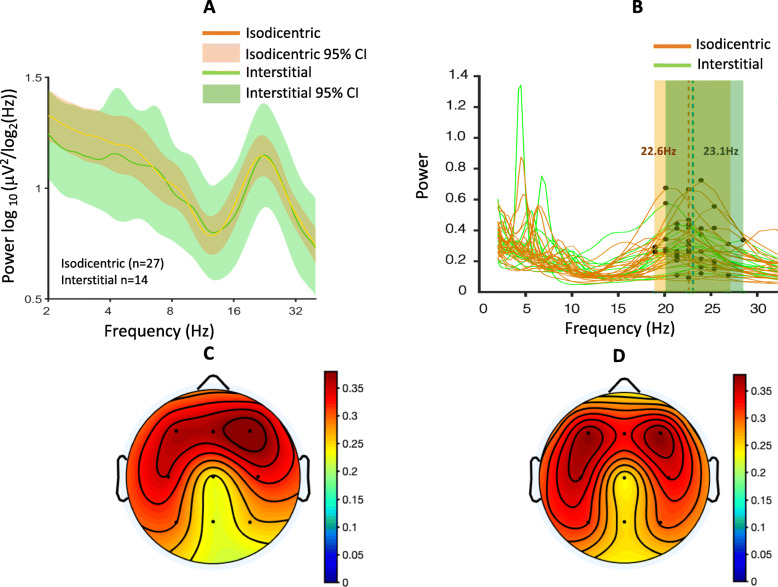
Duplication type and beta power/beta peak frequency. **a** Spectral profiles of isodicentric (orange) and interstitial (green) duplication groups. PSDs are averaged across channels, log10 transformed, and then averaged across participants; colored highlights represent 95% confidence intervals. **b** PSDs derived from isodicentric (orange) and interstitial (green) duplication groups. Beta peaks from each individual are labeled in black (group-level averages: isodicentric, *f* = 22.6 Hz; interstitial, *f* = 23.1 Hz.). **c** Mean topographic scalp power (mean across participants at the group-level peak frequency, *f* = 22.8 Hz) for participants with interstitial duplications. **d** Mean topographic scalp power (mean across participants at the group-level peak frequency, f = 22.8 Hz) for participants with isodicentric duplications

Neither beta power (*R*^2^ = 0.014, 95% CI − 0.41 to 0.19, *p* = 0.47) nor BPF (*R*^2^ = 0.005, 95% CI − 0.24 to 0.37, *p* = 0.65) correlated with age (Fig. 2a, b). There were no differences in beta power between duplication types (Fig. 3a; *R*^2^ = 5 × 10^−5^, 95% CI − 0.31 to 0.30, *p* = 0.96). BPF did not differ between duplication types. Individual beta band peaks derived from participants in the two duplication groups are shown in Fig. 3b, and the average peak frequency for the isodicentric and interstitial duplication groups were 22.6 Hz and 23.1 Hz, respectively. Mean topographic distribution of power across the scalp at the mean peak frequency is shown for each duplication type (interstitial, Fig. 3c, isodicentric, Fig. 3d). Both duplication types showed excessive beta power similar to that found in previous work [4, 5].

**Fig. 4:**
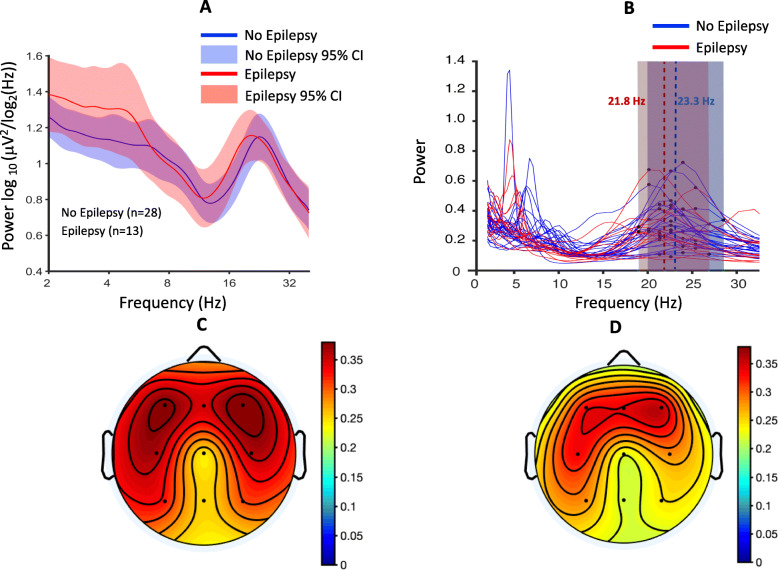
Epilepsy and beta power/beta peak frequency. **a** Spectral profiles of epilepsy (red) and non-epilepsy (blue) groups. PSDs are averaged across channels, log10 transformed, and then averaged across participants; colored highlights represent 95% confidence intervals. **b** PSDs derived from epilepsy (red) and non-epilepsy (blue) groups. Beta peaks from each individual is labeled in black (group-level averages: epilepsy, *f* = 21.8 Hz; non-epilepsy, *f* = 23.3 Hz). **c** Mean topographic scalp power (mean across participants at the group-level peak frequency, *f* = 22.8 Hz) in the epilepsy group. **d** Mean topographic scalp power (mean across participants at the group-level peak frequency, *f* = 22.8 Hz) in the non-epilepsy group

Beta power did not significantly differ between those with and without epilepsy (*R*^2^ = 3 × 10^−4^, 95% CI − 0.28 to 0.32, *p* = 0.90, Fig. 4a). However, BPF did significantly differentiate groups, with children with epilepsy showing a significantly lower peak frequency compared to those without epilepsy (correlation: *R*^2^ = 0.11, 95% CI:− 0.58 to − 0.02, *p* = 0.038; *t* test: *t* = 2.15, 95% CI 0.08–2.86, *d* = 0.07). Individual peaks captured for each participant in the beta frequency range are shown in Fig. 4b, with average peak frequency within the epilepsy and non-epilepsy groups being 21.8 Hz and 23.3 Hz, respectively. Mean topographic distribution of power across the scalp at the mean group-level peak frequency is shown for epilepsy and non-epilepsy groups in Fig. 4c and d.

### Study 2: Relation to cognition and adaptive skills

Behavioral testing is summarized in Table 1. Regression models to investigate predictors of beta power revealed that verbal and non-verbal cognition, and DLS, were not predictors of beta power (VDQ: *R*^2^ = 0.005, *p* = 0.68, NVDQ: *R*^2^ = 0.0002, *p* = 0.94, DLS: *R*^2^ = 0.0002, *p* = 0.97).

**Fig. 5:**
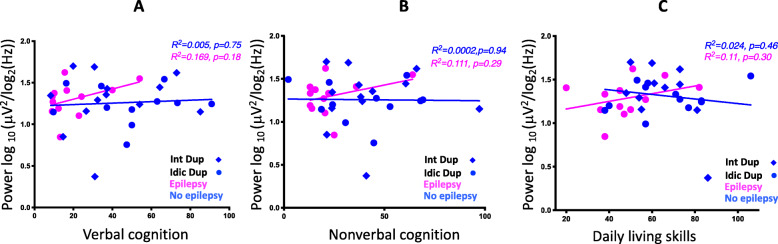
Cognition, daily living skills, and beta power. Beta power vs. verbal (**a**) and non-verbal cognition (**b**), and DLS (**c**). Participants with epilepsy are shown in pink and those without epilepsy in blue

We also performed regression models within the epilepsy and non-epilepsy groups separately and did not find meaningful associations (non-epilepsy group: VDQ: *R*^2^ = 0.005, 95% CI − 0.34 to 0.46, *p* = 0.75, NVDQ: *R*^2^ = 0.0002, 95% CI − 0.42 to 0.39, *p* = 0.94, DLS: *R*^2^ = 0.024, 95% CI − 0.53 to 0.26, *p* = 0.46; epilepsy group: VDQ: *R*^2^ = 0.17, 95% CI − 0.21 to 0.80, *p* = 0.18, NVDQ: *R*^2^ = 0.11, 95% CI − 0.30 to 0.76, *p* = 0.29, DLS: *R*^2^ = 0.11, 95% CI − 0.30 to 0.76, *p* = 0.30) (Fig. 5a–c).

**Fig. 6:**
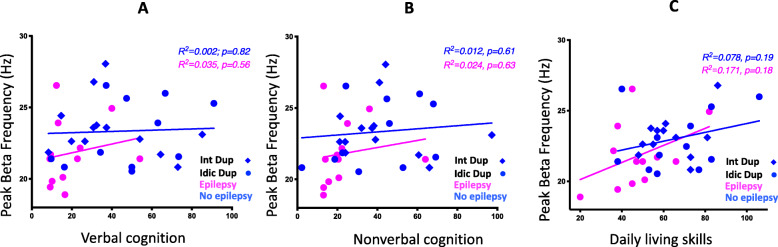
Cognition, daily living skills, and beta peak frequency. **a**, **b** Beta peak frequency vs. verbal and non-verbal cognition. **c** Beta peak frequency vs. DLS. Participants with epilepsy are shown in pink and those without epilepsy in blue

Neither verbal nor non-verbal cognition predicted BPF within the Dup15q syndrome cohort (VDQ: *R*^2^ = 0.049, *p* = 0.19, NVDQ: *R*^2^ = 0.055, *p* = 0.17). Upon performing regression models within the epilepsy and non-epilepsy groups separately, we found no significant relationship within the non-epilepsy group (non-epilepsy group: VDQ: *R*^2^ = 0.002, 95% CI − 0.36 to 0.44, *p* = 0.82, NVDQ: *R*^2^ = 0.012, 95% CI − 0.31 to 0.49, *p* = 0.61; epilepsy group: VDQ: *R*^2^ = 0.035, 95% CI − 0.43 to 0.69, *p* = 0.56, NVDQ: *R*^2^ = 0.024, 95% CI − 0.46 to 0.67, *p* = 0.63) (Fig. 6a, b). A moderate correlation between BPF and measure of DLS was seen, in that participants with lower adaptive skills had significantly lower peak frequency (*R*^2^ = 0.166 95% CI 0.09–0.65, *p* = 0.01). This relationship may be driven by individuals who have epilepsy, as participants with epilepsy have lower DLS scores and lower BPF [epilepsy group: mean DLS score = 47.9, mean BPF = 21.8 Hz] compared to those in the non-epilepsy group [non-epilepsy: mean DLS score = 65.0, mean BPF = 23.0 Hz]. Nonetheless, BPF accounted for a similar proportion of DLS variance in the epilepsy subgroup as in the overall cohort (epilepsy group: *R*^2^ = 0.17, 95% CI − 0.21 to 0.79, *p* = 0.18; non-epilepsy group: *R*^2^ = 0.08, 95% CI − 0.13 to 0.61, *p* = 0.19) (Fig. 6c), suggesting a possible correlation within the epilepsy subgroup that we were underpowered to detect.

### Study 3: Stability over time

**Fig. 7:**
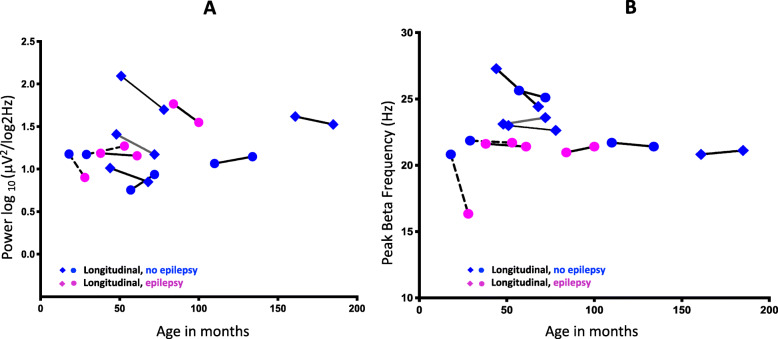
Longitudinal beta power and beta peak frequency. **a**, **b** Scatter plots of channel-averaged beta power and BPF derived from participants across multiple visits. Participants with epilepsy are shown in pink and those without epilepsy in blue. Longitudinal visits are connected by lines. Data connected with dotted lines represent participants that developed epilepsy between visits

The ICC derived from beta power from participants that had at least two EEGs was 0.93 (95% CI 0.63–0.98). ICC derived from BPFs from the same participants was 0.92 (95% CI 0.64–0.98), indicating moderate to excellent stability of both beta power and BPF across multiple EEG recordings (Fig. 7). Since two out of the ten participants developed epilepsy between visits, they were excluded from the ICC analysis.

### Study 4: Reproducibility from research to clinical EEG

**Fig. 8:**
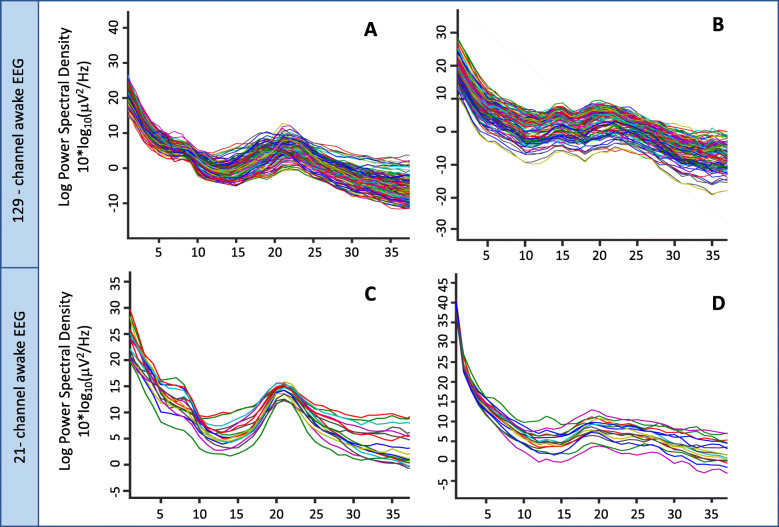
High- vs. low-density EEG PSDs. PSDs derived from research EEG (top row) and clinical EEG (bottom row) from two participants are shown. **a**, **c** PSDs from research and clinical EEG respectively of first participant. **b**, **d** PSDs from research and clinical EEG respectively of second participant. Individual channels are shown in different colors

The ICC derived from spectral power values computed from participants with high-density research and low-density clinical EEG recordings was 0.94 (95% confidence interval 0.60–0.98), indicating moderate to excellent reproducibility of the biomarker from research to clinical recordings. Figure 8 shows PSD plots of research EEGs (top row) and clinical EEGs (bottom row) of two representative participants.

## Discussion

Elevated beta band oscillations represent a robust, easily measurable biomarker of Dup15q syndrome, a genetic variant highly penetrant for NDDs and a promising target for future clinical trials, particularly disease modifying therapies. Here, we extended our quantification of this biomarker by testing properties that would inform its use in future trials, namely its relation to clinical and behavioral features, stability over time, and reproducibility across data collection systems and analytic pipelines. Key results included (1) differences in BPF between those with and without epilepsy, (2) stability over time based on consistency of signal between two EEG recordings at least 10 months apart, (3) reproducibility between research and clinical EEG recordings obtained as part of children’s routine epilepsy monitoring, and (4) reproducibility across two analytic pipelines that employed different frequency transforms and normalization.

### Beta oscillations in Dup15q syndrome

Spontaneous beta oscillations typically observed in human EEG reflect a state of cortical activation [41] through a network of inhibitory interneurons and pyramidal cells [42]. The period (and thus frequency) of neural oscillations is determined in part by the time constants on postsynaptic receptors (i.e., faster time constants yields faster oscillatory frequencies), as is known to be true of gamma oscillations and GABA_A_Rs [43]. To this effect, barbiturates such as phenobarbital and pentobarbital increase the duration of GABA_A_R channel opening [44, 45], as does zolpidem, a benzodiazepine-like compound (described as a “benzodiazepine agonist” in older literature) [46, 47]. Moreover, benzodiazepines decrease the frequency of beta oscillations [42, 47], lending plausibility to the idea that increases in beta power observed with both pharmacological GABA_A_R modulation and 15q duplication result from shifting of fast oscillations towards a resonate frequency in beta (i.e., the frequency the system prefers to oscillate at when energy is added), thus explaining the very large amplitude of beta seen in both contexts. This mechanism is speculative, and it remains unknown how it would interact with other factors (e.g., epilepsy and receptor properties altered by antiepileptic medication). Nevertheless, based on the hypothesized role of GABA_A_Rs in the generation of these oscillations, the EEG biomarker in Dup15q syndrome could enrich clinical trials by serving as a measure of drug target engagement or as a proximal outcome measure that precedes behavioral responses to pharmacological treatments that modulate GABAergic neurotransmission.

### Beta oscillations and relationship to phenotype

We found no significant relationship between beta parameters and age, demonstrating that beta power and frequency are likely readouts of the fundamental disease pathology in Dup15q syndrome that remains unchanged over development. There is increased interest in identifying biomarkers of NDDs that relate to clinical symptomatology. We found that the strongest clinical predictor of the EEG signature in Dup15q syndrome was epilepsy, as BPF differed based on epilepsy status.

As in many syndromic NDDs, epilepsy in Dup15q syndrome is associated with greater functional impairment [48]. We urge caution in not overinterpreting the relationship between epilepsy and beta oscillations, as without preclinical models to manipulate the underlying altered circuitry, we will not be able to prove directionality of the association. Epilepsy (both active seizures and interictal epileptiform EEG activity) causes significant changes in the EEG, such as slowing of background oscillations and spike/wave discharges, and these changes have been demonstrated in overnight EEG studies of patients with Dup15q syndrome [49]. Antiepileptic medications also change oscillatory activity, with some medications, such as GABA_A_R positive allosteric modulators, causing further elevation of high frequency oscillations, while others, such as phenytoin or carbamazepine, increasing generalized cortical slowing. If and how seizures and/or their treatments slow the GABA_A_R time constant to produce a slower beta frequency is not fully understood. This question may be best addressed in preclinical models, in which various aspects of the circuits can be directly manipulated. However, from a clinical perspective, this association has tremendous promise, and future studies will assess whether BPF serves as a predictor of epilepsy early in development or as an informative marker of response to antiepileptic drug studies.

We found no correlation between duplication type and beta power or BPF. This negative result should be interpreted with caution: The number of individuals with EEG and clinical data is limited, which enforced (1) combining different clinical assessments of cognition (MSEL, Differential Ability Scales), (2) combining a broad age range, (3) combining both genotypes (interstitial, isodicentric), and (4) combining individuals with and without epilepsy. It is possible that future analyses, possibly with more participants, that account for those sources of variance will find a relation. However, it is also well possible that a relation between EEG Beta oscillations does not exist. Beta oscillations are an emergent network property that may non-linearly increase and then asymptomatically saturate based on GABA_A_R expression. In Dup15q syndrome (both interstitial and isodicentric), such a saturation may be reached (compatible with finding no higher beta in isodicentric duplications despite a presumably higher gene-dose effect). Therefore, there may not be room to measure inter-subject variability that could correlate with symptom severity. Thus, even in the absence of a correlation with symptom severity, elevated beta oscillations in Dup15q syndrome could still be a useful biomarker of altered GABAergic neurotransmission that could be used in drug development. The saturation of beta oscillations based on GABA_A_R expression could, for instance, be explained biophysically by the short duration of the beta cycle (33–83 ms), which limits the number of neurons that can be recruited for the oscillation [50]. Future studies with computational models backed by experiments will be needed to assess changes of individual cellular biophysical parameters to network oscillation.

We found no correlation between behavior and beta power or BPF in Dup15q syndrome. Although BPF was associated with adaptive skills, this relationship may be driven by epilepsy status, as children with epilepsy have lower overall adaptive skills compared to those without epilepsy [51–54]. It is possible that our behavioral measures lack sufficient sensitivity to capture the range of clinical variability in individuals with Dup15q syndrome. However, our results build on findings in Angelman syndrome showing that children with 15q11-q13 deletions (deletion Angelman) have lower beta (23 Hz) power than children with other etiologies that principally affect *UBE3A* (nondeletion Angelman) [24]. In fact, deletion and nondeletion Angelman differ not only in beta power but also in clinical severity [55–58]. Given that beta power and clinical severity covary in Angelman, it is possible that a similar relationship exists in Dup15q syndrome, but that either the severity of cognitive impairment in this population or other limitations to the psychometric properties of the tests used in this sample may limit our ability to capture subtle relationships between this biomarker and behavior. However, this lack of correlation with behavior does not undermine its potential for use as a marker of drug target engagement in clinical trials, as here we did not test whether change in beta oscillations predict or relate to change in clinical outcomes.

### Stability and reproducibility of the EEG biomarker

Our data show stability across multiple recordings and reproducibility across data acquisition (research vs clinical EEG) methods. A fundamental question addressed by our study was whether this biomarker could be quantified from low-density clinical recordings performed outside of research study setting, which is of particular importance given that most of these children undergo clinical EEGs regularly as part of their clinical monitoring. One of the biggest challenges in EEG studies in NDDs is data collection itself (i.e., bringing in participants to sites to collect the research EEG). Future efforts that would bypass data collection in an expensive, structured research setting and quantify beta power and frequency in a repository of clinical EEGs would allow additional analyses to be far more clinically relevant, scalable, and statistically powered. In fact, our study has already motivated these analyses in an ongoing clinical trial for epilepsy in Dup15q syndrome, with beta power being quantified through clinical EEG collected at baseline.

### Biomarkers in neurodevelopmental disorders

Research in preclinical models has truly advanced our insights into the pathological mechanisms underlying various NDDs. Despite this knowledge, even presumably well-designed clinical trials have struggled to demonstrate significant effects in treatment groups compared to placebo [59, 60]. These challenges may reflect several gaps: (1) even when the treatment has a clear biological, mechanistic target, lack of measurement of drug target engagement limits the ability to determine if a drug could have an effect; (2) in the setting of the biological and developmental heterogeneity of these conditions, there are few objective methods for patient selection for trials; (3) outcome measures themselves often prove ineffective to capture the effect of a treatment because of insensitivity to short term change or vulnerability to reporting bias or placebo effect; and (4) outcome measures chosen based on preclinical research do not translate to meaningful or modifiable patient-centered outcomes. The identification and quantification of objective biomarkers can mitigate some of these challenges by facilitating patient stratification, measuring drug target engagement, and defining outcomes relatively resistant to the placebo effect.

As EEG can measure circuit-level treatment response before behavior changes can be observed, EEG biomarkers have the potential to address the challenges involved in pharmacological trials [60]. To that end, this study holds promise in identifying EEG biomarkers in a rare genetic population highly penetrant for NDDs. The lack of a detectable association between the EEG biomarker and age or symptom severity in this sample demonstrates that this biomarker likely relates to the underlying neurobiology in Dup15q syndrome, with possible relation to GABAergic dysfunction. The hypothesis that we can quantitatively measure the impact of altered GABA signaling is particularly exciting in the field of NDDs, as dysfunction in the dynamics of cortical GABAergic circuitry may be implicated in syndromes other than Dup15q syndrome. Furthermore, converging evidence from gene linkage studies suggest that point mutations in the GABA_A_ β3/α5/γ3 gene cluster may also be implicated in other NDDs [7, 61, 62]. It is possible that elevated beta oscillations in individuals with other NDDs may herald other genetic causes of altered GABA neurotransmission, such as point mutations in the GABA_A_ β3/α5/γ3 gene cluster. Future studies can therefore leverage existing electrophysiological data from children with NDDs and explore the utility of the EEG biomarker in Dup15q syndrome to predict genetic variants in children with NDDs and further our understanding of underlying circuit-level pathology in a subset of these children.

### Limitations, conclusions, and future directions

Our work herein established the robustness and reproducibility of the EEG beta phenotype as a biomarker of Dup15q syndrome. Studies of rare genetic disorders are often limited by small sample number, and we also faced these sample size challenges, particularly in the studies of stability over time and reproducibility. Findings from our reproducibility study have led to the development of a new data acquisition and storage pipeline for clinical overnight sleep EEG recordings of children with Dup15q syndrome across the world, in partnership with the Dup15q Alliance. We will utilize these recordings to investigate the presence of beta oscillations in sleep EEG and to characterize sleep physiology in children with the syndrome. As more children with syndromic forms of NDDs undergo clinical EEG investigation, this pipeline will directly guide decisions to replace research EEG recordings with clinical ones, thereby facilitating larger scale studies of EEG biomarkers across syndromic NDDs.

## Data Availability

EEG data from Dup15q syndrome children are available from the corresponding author on reasonable request.

## Abbreviations

Dup15q syndrome: Duplication 15q11.2-q13.1 syndrome
NDD: Neurodevelopmental disorder
EEG: Electroencephalogram
ASD: Autism spectrum disorder
GABA_A_: Gamma-aminobutyric acid type A
ID: Intellectual disability
UCLA: University of California, Los Angeles
DQ: Developmental quotient
VABS: Vineland Adaptive Behavior Scale
DLS: Daily living skills
BPF: Beta peak frequency
IRB: Institutional review board
ICA: Independent component analysis
ICC: Intraclass correlation coefficient
PSD: Power spectral density
